# Push-Out Bond Strength and SEM Evaluation in Roots Filled with Two Different Techniques Using New and Conventional Sealers

**DOI:** 10.3390/ma11091620

**Published:** 2018-09-05

**Authors:** Pervin Dabaj, Atakan Kalender, Ayce Unverdi Eldeniz

**Affiliations:** 1 Department of Endodontics, Faculty of Dentistry, Near East University, 99258 Lefkosa, Mersin 10, Turkey; atakan.kalender@neu.edu.tr; 2 Department of Endodontics, Faculty of Dentistry, Selcuk University, Konya 42130, Turkey; ayce71@hotmail.com

**Keywords:** push-out test, bond strength, obturation materials, Calamus Flow Delivery System, Endosequence-BC-Sealer

## Abstract

The aim of this study was to evaluate the influence of calcium-silicate-based sealer (Endosequence-BC-Sealer) in roots, filled with thermo-plasticized injectable technique aided by Calamus-Flow-Delivery-System, on bond strength to radicular dentin, in comparison with conventional epoxy-resin-based sealer (AH-Plus) along with cold-lateral-compaction technique. Root canals of mandibular-premolar teeth (*n* = 80) were instrumented using Protaper Universal rotary files and were randomly divided into four experimental groups (*n* = 20) as follows: (1) AH-Plus + cold-lateral-compaction technique; (2) Endosequence-BC-Sealer + cold-lateral-compaction technique; (3) AH-Plus + thermo-plasticized injectable technique; and (4) Endosequence-BC-Sealer + thermo-plasticized injectable technique. Horizontal disc shaped samples from each group (*n* = 60/group) were obtained and push-out bond strength testing was performed at a cross-head speed of 0.5 mm/min. Data were analyzed statistically using nonparametric Kruskal-Wallis analysis and Mann-Whitney test (*p* < 0.001). The statistical analysis revealed a significant difference amongst the groups (*p* < 0.001). The highest bond strength values were found in group 1 compared with all the other experimental groups (*p* < 0.001), whereas the lowest bond strength values were found in group 4 (*p* < 0.001). It was concluded that thermo-plasticized injectable technique with Calamus-Flow-Delivery-System lowered the bond strengths of the sealers, especially Endosequence-BC-Sealer. Therefore, this technique is not recommended to calcium-silicate-based sealers. Further studies are needed to confirm the findings of this study.

## 1. Introduction

For a hermetic seal, sealers are required to adhere to both the radicular dentin and the core filling [1], while avoiding disruption of the root canal system for a long term and resisting dislocation during tooth flexure and operative procedures [2]. Without a sealer it is not possible to assure a desirable seal of a root canal since gutta-percha alone cannot provide a complete seal by adhering to the dentinal walls thus causing a failure in the sealing quality [3].

In endodontics, sealers are classified on the basis of their main chemical components. Many different types of sealers are available on the market, based; zinc oxide, calcium hydroxide, glass ionomer, epoxy resin, silicone and methacrylate [4]. AH Plus (Dentsply DeTrey GmbH, Konstanz, Germany) is an epoxy-resin based sealer and like others of its type has been commonly used for many years owing to its adequate radiopacity, flow, dimensional stability, low solubility and low concentration, and high resistance [5]. It has shown higher bond strength to dentin than zinc oxide, calcium hydroxide and glass-ionomer sealers [3,6].

Recently, a bioceramic based sealer known as Endosequence BC Sealer (Brasseler USA, Savannah, GA, USA) (also known as iRoot SP Injectable Root Canal Sealer, Innovative Bioceramix Inc., Vancouver, BC, Canada) has been introduced to the market and is regarded as an efficacious technology. Unlike other conventional sealers, the premixed and ready-to-use Endosequence BC Sealer, which is composed of calcium phosphate, calcium silicate, calcium hydroxide, zirconium oxide, filler and thickening agents [7], is an injectable sealer with its disposable delivery tips for the intracanal delivery of the material. With its hydrophilic characteristics, it utilizes moisture to initiate and complete its setting reaction [8]. Once setting occurs, the result is a chemical bonding with a void-free interface between the gutta-percha, sealer and radicular dentin [7]. As stated by the manufacturer, it is a highly biocompatible sealer (even more biocompatible than AH Plus) with antibacterial qualities, due to its highly alkaline pH [9].

Many factors can affect the adhesion of a root canal sealer one of which is the presence of the smear layer. None of the numerous studies regarding the effects of this layer on adhesion and microleakage confirms whether or not the removal of smear layer impacts the sealing ability [4]. Nonetheless, there are studies showing that the presence of this layer is a negative factor in root canal sealing since an interface is formed between the sealing material and the radicular dentin which reduces adhesion. Therefore, most studies recommend the removal of the smear layer to aid the obturating material’s adaptability to the root canals [10,11]. On the other hand, the bond strength of some sealers to radicular dentin, however, was improved in the presence of the smear layer [4].

The popularity of the thermo-plasticized injectable technique is increasing owing to its easy adjustment to root canal complexities. Because it forms a thicker filling that prevents scanty formation and a three-dimensional sealing of the root canal system is obtained [12]. To the best of our knowledge, little is done regarding the literature on the effectiveness of thermo-plasticized injectable technique using Endosequence BC Sealer with the aid of Calamus Flow Delivery System (Dentsply, First Edition, Woodinville, WA, USA) on the push-out bonding strength of endodontically treated teeth. Therefore, this study aimed at evaluating and comparing the push-out bond strengths of root canals obturated with Endosequence BC Sealer, along with the thermo-plasticized injectable technique, while comparing it against AH Plus, and cold lateral compaction technique. The null hypothesis tested was that Endosequence BC Sealer along with the thermo-plasticized injectable technique produces better push-out bond strength values than AH Plus and cold lateral compaction technique.

## 2. Materials and Methods

### 2.1. Sample Selection and Sample Size Calculation

This study was approved by the Scientific and Ethics Commission of the Near East University (protocol #2016/37-286). A total number of 80 single-rooted mandibular premolars with fully developed apices and roots with curvature angle less than 10 degrees (mild curvature), as determined by the Schneider’s method [13], were used. In the experimental investigation, the informed consents of all human subjects participating were obtained. Pairs of similar teeth, scheduled for extraction because of periodontal disease, caries or orthodontic reasons, were selected. There was no available data regarding age and gender. Mandibular premolar teeth were chosen for the study as they generally have oval shaped root canals; however, in order to verify this, and to measure the sample size, a series of conventional bucco-lingual, and mesio-distal periapical radiographs were taken and gone under a visual inspection. Teeth with previous endodontic treatment, having more than one root canal, with calcified root canals or fractured roots, and internal or external root resorption and curvature or open apices were excluded. After the extraction process, all teeth were stored in formalin. In the preparation process, the teeth were all diligently cleared off from their soft and hard tissues on roots using scalpel blades. The teeth were disinfected by keeping them in 5.25% NaOCl solution for 2 h after which they were thoroughly rinsed under running tap water. They were then placed in sterile distilled water until further use.

### 2.2. Root Canal Preparation

The crowns of the samples were removed at or below the cemento-enamel-junction (CEJ) by using a water-cooled diamond saw at low speed and a standardized root length of about 15–16 mm was created. The lengths of the samples were measured starting from apex to CEJ using a manual caliper. A number 10 K-file (Dentsply Maillefer, Ballaigues, Switzerland) was inserted into the canal until the tip was barely visible beyond the apex ensuring apical patency. The working length was then determined after subtracting 1 mm from this length. Cleaning and shaping were all done with a crown-down instrumentation technique using Protaper Universal rotary files (Dentsply Maillefer, Ballaigues, Switzerland) compatible with the manufacturer’s instructions. All instruments were used at a 16:1 gear reduction hand-piece powered by a torque-controlled electric motor (X-smart; Dentsply Maillefer, Ballaigues, Switzerland) at a constant rotation of 250 rpm. All samples were prepared using a sequence of SX, S1, S2, F1, F2, F3 and F4 as a final apical file applying in-and-out motion, so that the volumes of all canals were standardized to the same taper. Before insertion into canals, 2% Gluco-CheX gel (Cerkamed, Stalowa Wola, Poland) was used on each file. After the use of each instrument, all debris, including the smear layer, were removed by 5.25% NaOCl and 17% EDTA (ethylenediaminetetraacetic acid) as a final irrigant for 1 min, and were then rinsed off with sterile distilled water to remove residues of the solutions. The root canals were then properly dried with sterile paper points (Dentsply Maillefer, Ballaigues, Switzerland).

### 2.3. Root Filling

The samples were divided randomly into 4 groups (*n* = 20), and obturated as follows.

Group 1 (AH/CLC): Using a cold lateral compaction technique (CLC), each canal was obturated with gutta-percha cones and AH Plus (AH) sealer. The master gutta-percha cone (Dentsply Maillefer) (size 40) was coated with a sealer prepared by mixing into a thickness recommended by the manufacturer. It was then inserted into the canal until a tug-back at a working length was obtained. Lateral compaction was achieved in each canal using fine gutta-percha accessory cones (Dentsply Maillefer) (size 20) coated lightly with sealer and finger spreader (Thomas, Bourges, France) (size 20) that initially reached within 2 mm of the full working length. The procedure was repeated till the spreader could no longer be inserted for more than 2 mm into the canal orifice. Excess gutta-percha was removed by a heated instrument until it was 0.5 mm below the orifice and compressed towards the apical direction using a cold plugger size 8 (0.8 mm diameter, Dentsply Maillefer) to compact the gutta-percha in the coronal portion of the root canal.

Group 2 (ES/CLC): By CLC technique, each canal was obturated with gutta-percha cones and Endosequence BC Sealer (ES). The sealer was placed into canals using disposable intracanal tips (Brasseler, USA). The tip was placed into the canal no deeper than the middle one third and the sealer was injected by compressing the plunger of the syringe. A master gutta-percha cone (size 40) was placed at the working length. The fine accessory gutta-percha cones (size 20) were coated with the sealer which was also injected onto a sterile mixing glass then inserted into canals using a finger spreader (size 20), and compressed until the root canals were completely filled. Excess gutta-percha was then removed and compressed with a cold plugger (size 8).

Group 3 (AH/C): Using thermo-plasticized injectable technique (C) by Calamus Flow Delivery System (Dentsply-Tulsa Dental, Tulsa, OK, USA) the canals were obturated with AH Plus sealer. The canals were coated with the sealer using a lentulo spiral (Mani Paste Carriers, Tochigi, Japan), and a master gutta-percha cone (size 40) was inserted into the canal at working length. Master cone was seared off at the level of orifice using the electric heated plugger tip (large-taper 0.06) of the system. The cooling instrument was removed from the canal, bringing out an increment of the thermo-softened gutta-percha after deactivating the heated plugger tip. To compact the gutta-percha into the coronal portion of the canal, a vertical force was applied with a size 11 plugger (1.1 mm in diameter). This procedure was repeated twice, first at a level of 3–4 mm deeper than the orifice (by the aid of the electric heated plugger tip size middle-taper 0.05) and again vertically condensing the gutta-percha in the middle portion of the canal by size 8 plugger (0.8 mm diameter) and second to a level of 4 mm of working length (by the aid of the electric heated plugger tip size small-taper 0.03) and vertically condensing the gutta-percha in the apical portion of the canal using size 7 plugger (0.7 mm diameter). The reason of doing so was to create a dense apical plug. The rest of the canal was obturated by the backfilling of thermo-softened gutta-percha heated at 180 °C to optimally fill the canal. This was achieved by injecting warm gutta-percha, using the electric gutta-percha cartridge 20 G (0.8 mm diameter), each time injecting 3–4 mm segments and condensing the gutta-percha vertically with a plugger (size 8).

Group 4 (ES/C): The canals, as in group 3, were obturated with Endosequence BC Sealer using thermo-plasticized injectable technique. The sealer was injected into canal and a size 40 master cone was inserted according to the working length. Master cone was again seared off three times until an apical barrier was accomplished. Remaining canal space was backfilled with thermo-softened gutta-percha, incrementally, and condensed coronally.

For each sample periapical radiographs were taken right after the obturation procedures in order to verify the radiographic quality of the obturation. It is of high importance to keep the roots moist during the whole experiment by wrapping them in gauze moistened in sterile distilled water. Excess core material and sealer were carefully removed using sterile scalpels, and the root surfaces were wiped with gauze and ethanol. No temporary or permanent restoration was placed over the obturating material. All groups were kept in an incubator, and allowed for the full setting of the sealers for 7 days at 37 °C and 100% humidity (full setting time; AH Plus 8 h and 25 min and Endosequence BC Sealer 168 h as stated by the manufacturers). 

### 2.4. Sample Preparation for Push-Out Test and Scanning Electron Microscope (SEM) Analysis

Samples were perpendicularly sectioned along the long axis using a water-cooled diamond blade on an Isomet precision cut-off machine (IsoMet 1000, Buehler, Lake Bluff, IL, USA), with a constant rotation of 150 rpm. Three 1 mm thick horizontal disc shaped slices were created from each specimen from coronal, middle and apical thirds. By doing so, a total of 60 slices per group were obtained (*n* = 60/group). By a manual caliper each slice was measured in thickness. Two random samples (1 from coronal third and 1 from middle third) from each group were selected for SEM (EVO LS 10, Zeiss, Germany) evaluation before the push-out test.

### 2.5. Push-Out Test

A Universal Testing Machine (EZ 50, Shimadzu, Kyoto, Japan) was used evaluating bond strengths. The root slices were mounted on an acrylic block of 2 × 2 cm in dimension. In order to provide the most extended coverage over the filling material, all samples were loaded using a 0.5 mm (for the apical third samples), 0.8 mm (for the middle third samples), and 1 mm (for the coronal third samples) diameter stainless steel cylindrical plungers avoiding contact with the canal walls. All loading was administered in an apical-coronal direction towards the larger part of the slice averting any limitations to the movement of the filling material at a cross-head speed of 0.5 mm/min until bond failure attained.

The bond strength was expressed in MPa, the load at the failure was recorded in Newtons and was divided by the area of the bonded interface, and was calculated by the following formula: A = 2πr × h, where π is the constant 3.14, r is the root canal radius, and h is the thickness of the slice in millimeters.

### 2.6. SEM Analysis

Slices which were moistened with distilled water were sandpapered and debris created at the end of the procedure was removed by an ultrasonic bath (Bandelin, Sonorex Digitec, Germany) for 10 min. Slices were conditioned using a 5 mol HCl for 45 s after which all the organic debris was removed, and then rinsed off using distilled water to remove any remaining residue of the solution. The conditioning was repeated the second time using 2.5% NaOCl for 10 min which would help expose resin tags and again washed with distilled water. With 50% ethyl alcohol the washed slices were dehydrated, mounted on stubs, sputter coated with gold, and examined under SEM. Photomicrographs were taken from the slices.

### 2.7. Statistical Analysis

Statistical analysis was performed with nonparametric tests by Kruskal-Wallis analysis for overall comparisons and Mann-Whitney test for multiple comparisons, with significance set as *p* < 0.001 and a level of confidence set as 95%.

## 3. Results

Table 1 represents the mean push-out bond strength values and standard deviations for all groups. According to the Kruskal-Wallis analysis, there were significant differences among the groups (*p* < 0.001). The values of mean bond strengths ranged from 3.1267 MPa (Endosequence BC Sealer-thermo-plasticized injectable technique) to 17.48 MPa (AH Plus-cold lateral compaction technique). This revealed the highest bond strength value group as in group 1 (AH Plus-cold lateral compaction) compared with all the other groups (*p* < 0.001). In bond strength, group 4 (Endosequence BC Sealer-thermo-plasticized injectable technique) and group 2 (Endosequence BC Sealer-cold lateral compaction) showed no notable difference from each other (*p* > 0.001), on the contrary, when compared with the other experimental groups, they both had lower bond strength values. Group 3 (AH Plus-thermo-plasticized injectable technique) had the second highest bond strength (*p* < 0.001). Mann-Whitney test also showed that there were significant differences (*p* > 0.001) at all levels (Table 2). The coronal levels of all groups displayed the highest mean push-out bond strength values. There were no significant differences between groups 2 and 4, at all levels. Group 1, on the contrary, showed significant differences at all levels (*p* > 0.001) as compared with any other experimental groups.

## 4. Discussion

In static and dynamic situations, the integrity of a root canal seal depends greatly on the adhesion of the root canal filling material to radicular dentinal walls [14]. In a static situation, in order to prevent the percolation of fluids between the filling, and the radicular wall, the filling material should eliminate any space, thus preventing the accumulation of both stagnant tissue fluid, and microorganisms which may cause periapical disease [15]. In a dynamic situation, on the other hand, resistance to dislodgement of the filling during subsequent manipulation is of essence [16]. In order to strengthen the restored tooth and provide a better resistance to root fracture, increasing adhesive features to radicular dentin is a key resulting in the clinical longevity [15].

Amongst the many methods for measuring the adhesion qualities of root canal fillings, none has yet been accepted on a large scale [17]. However, the most effective method is the bond strength testing. This test of course is of no match to any clinical performance, and is not able to show any correlation between bond strength and clinical success. However, it gives substantial information by comparing different sealers and/or obturation techniques [18]. Push-out test was the chosen method for this study as this method is widely accepted, and used for recording the interfacial bond strengths of root canal filling materials and/or techniques to radicular dentin, even at low levels [2,19].

All groups in the present study showed measurable adhesive properties. Interestingly, the two groups with calcium-silicate-based sealer exhibited distinctively lower bond strengths. However, statistically there was no significant difference between the two groups. Therefore, the null hypothesis was clearly rejected. The reason why could be the use of traditional gutta-percha cones that prevented adhesive bonding between the core material and the sealer, resulting in debonding of the filling from the radicular dentin [20]. The use of Bioceramic cones, ActiV GP or C-Point might have helped to increase the bonding strength. C-Point with Endoequence BC Sealer, according to Pawar et al. [21] is claimed to push the sealer radially, enabling a more intimate adaptation with the irregular spaces of canal walls.

The findings of this study correlate with that of other studies. Gurgel-Filho and Martins [22] and Ungor et al. [16] utilized the similar test design and found higher bond strengths in teeth obturated with gutta-percha and AH Plus sealer. In the present study, AH Plus showed significantly higher bond strength than Endosequence BC Sealer when used in cold lateral compaction. An explanation for this result could be the formation of a covalent bond when diepoxide compounds and polyamine paste are mixed during the manipulation of epoxy-resin based sealers producing a reaction between each amine group in the collagen network with an open epoxide ring that would create a rigid and strong cross-linked polymer [23]. Moreover, other high-quality properties of epoxy-resin based sealers might also play an important role for higher bond strength including low shrinkage during setting, longer polymerization period and penetrating easily into micro-irregularities, due to their creep capacity and long-term dimensional stability [24].

**Figure 1:**
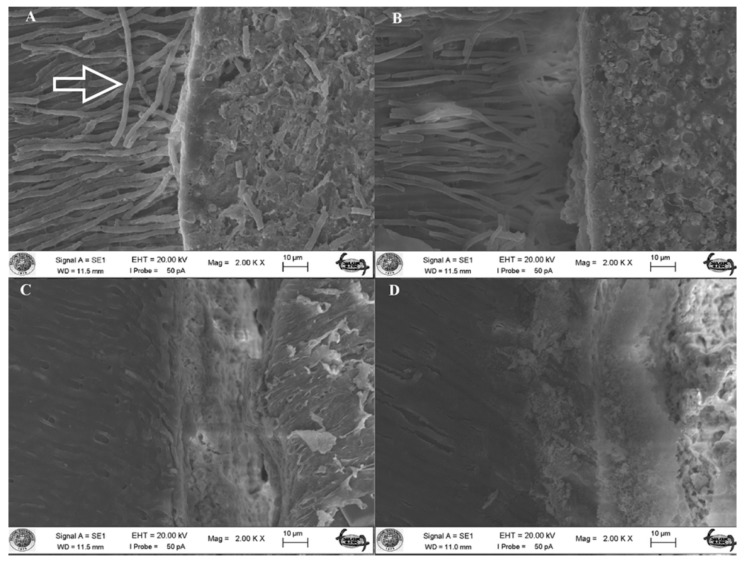
(**A**) Scanning electron micrograph (×2000) of a sample in group 1 (AH/CLC). Radicular dentinal wall is densely covered with AH Plus sealer and resin tags (indicated with an arrow) occluding the dentinal tubules; (**B**) Scanning electron micrograph (×2000) of a sample in group 3 (AH/C). Dentinal wall is partly covered with the sealer and tags protruding from the sealer; (**C**) Scanning electron micrograph (×2000) of a sample in group 2 (ES/CLC). The area is covered with the sealer but there is no visible sealer tags in dentinal tubules; (**D**) Scanning electron micrograph (×2000) of a sample in group 4 (ES/C) where there is almost debonding of the sealer from the radicular dentinal wall and no sign of tag elements.

During chemo-mechanical preparation, a layer of debris, the smear layer, is formed. This layer consists of organic and inorganic substances which include microorganisms and necrotic tissues occluding the radicular dentinal tubules [25]. Current theories regarding dentin bonding mechanisms offer to chemically modify the smear layer and directly bonding to it, or bonding to subjacent tooth structures after the removal of this layer [16]. When this layer is removed, a large number of dentinal tubules are exposed thus increasing the contact area, and the wettability resulting in a better adaptation of the sealer to radicular dentin by forming sealer tags [26]. The present study confirms these findings. SEM micrographs, only after removing the smear layer, showed penetration of sealer (resin) tags with the AH Plus groups (group 1 and 3) (Figure 1). This result is in accordance with Eldeniz et al. [27], who showed that AH Plus yielded a stronger bond to radicular dentin after removing the smear layer. However, when the smear layer is left intact, various sealers show a better bonding to the dentin because this layer contains moisture, and might possibly acts as a coupling agent by improving the adaptation quality of hydrophilic materials to the root canal walls [28,29]. Endosequence BC Sealer is a hydrophilic material, and utilizes the moisture in the smear layer—thus creating hydroxyapatite-like precipitation while setting, and adheres to dentin chemically [8]. Therefore, removal of this layer could be a less favorable process and could create a negative effect on the adaptation of this sealer to the canal walls. The moisture in the dentinal tubules may not be sufficient to help set the material causing lower bond strength of the sealer in root canals dried before obturation [30]. Therefore, such mechanical interlocking, was not the result of the Endosequence BC Sealer groups (group 2 and 4) in the current study (Figure 1). Related literature supports these findings by showing that the removal of the smear layer caused significantly less adhesion and more microleakage [29,31,32]. Moreover, the opened dentinal tubules may act as stress raisers, causing failure in the adhesive joint, for which the smear layer is rich in calcium and phosphate, therefore potentially promotes strong adhesion [33], and a change in the proportion of Ca:P in the tooth structure influences the adherence of calcium-silicate-based sealers, that need calcium ions in the dentin for biomineralization process [34].

The present study also investigated the effect of root-thirds on bond strength. In all experimental groups, the bond strength values were increased from apical to coronal direction, and revealed a significant difference. This could be explained by apical radicular dentin having a lesser number of tubules, thus a reduced sealer area, than the coronal part. The coronal part of the root canal system has a more complex tubular structure (higher tubular density/diameter), and apparently yields a better infiltration compared to its sclerotic apical counterpart. This agrees with the findings of Carneiro et al. [35] and Nagas et al. [36].

Warm obturation techniques, like thermo-plasticized injectable technique, that show to effectively fill in canal irregularities, and form a thicker filling avoiding scanty formation are popular, especially among endodontists [12]. Nonetheless, they come with several disadvantages as the current studies revealed that techniques can affect the adhesion quality of the sealer either physically or chemically, thus resulting in lower bond strength values. Looking at the findings of this study, with respect to the thermo-plasticized injectable technique, it would be appropriate to state that the adhesive failures happened more in Endosequence BC Sealer in group 4 than in AH Plus in group 3. This may be due to increased polymerization of the sealer [37]—since heat can accelerate hydration and hydroxyapatite formation reactions. Calcium-silicate-based sealers display a faster setting time [38] and their flowability rate decreases as reported by Camilleri et al. [39]. Therefore, the rapid setting causes increase in stiffness resulting in a lower bond strength. The intermediate bond strength established with AH Plus in group 3—however, this may be explained by AH Plus being a heat tolerant sealer, meaning warm obturation techniques do not affect its setting time and bonding quality as much as calcium-silicate-based sealers [12].

## 5. Conclusions

Taking the limitations of this in vitro study into account, the following conclusions can be obtained:

(1) AH Plus showed significantly higher push-out bonding strength amongst all experimental groups, regardless of the obturation techniques used.
(2) AH Plus, when used especially in cold lateral compaction technique, significantly proved to show the highest push-out bonding strength values amongst all experimental groups.
(3) Endosequence BC Sealer proved to be weaker in bonding strength when compared to AH Plus sealer, regardless of the obturation techniques used.
(4) The bonding strength of Endosequence BC Sealer is even more weakened in-conjuction-with thermo-plasticized injectable technique with Calamus Flow Delivery System—however, there was no significant difference between the two Endosequence BC Sealer groups.

## Figures and Tables

**Table 1:** Mean push-out bond strengths (MPa) and standard deviations for the experimental groups.

Group	Formulation	Mean Bond Strength	Standard Deviation
**1**	CLC-AH	17,48	7,131
**2**	CLC-ES	3,56	1,703
**3**	C-AH	9,573	7,291
**4**	C-ES	3,127	2,286

**Table 2:** Descriptive statistics of mean push-out bond strengths (MPa) and standard deviations at three levels for all groups.

Group	Level	Number	Mean Bond Strength	Standard Deviation
1 (CLC-AH)	Coronal	20	20,500	2,121
	Middle	20	19,467	10,619
	Apical	20	15,480	6,070
2 (CLC-ES)	Coronal	20	4,350	1,974
	Middle	20	3,250	1,768
	Apical	20	2,888	1,037
3 (C-AH)	Coronal	20	13,800	10,391
	Middle	20	13,400	4,243
	Apical	20	5,975	3,208
4 (C-ES)	Coronal	20	3,460	2,862
	Middle	20	3,186	1,508
	Apical	20	2,500	0,707
